# Supplementary material for: Effect of acupuncture on patients with major psychiatric disorder and related symptoms caused by earthquake exposure: Protocol for a scoping review of clinical studies
Source: PLoS One. 2023 Jan 27;18(1):e0281207. doi: 10.1371/journal.pone.0281207 (PMC9882882; doi:10.1371/journal.pone.0281207)
Supplement: S1 Table — (DOCX) [file pone.0281207.s001.docx]

**S1 Table. Search terms used in each database.**

**Medline via PubMed**

|  | Searches | Results |
| --- | --- | --- |
| #1 | (“earthquake"[MeSH] OR earthquake[Title/abstract] OR “tsunami"[MeSH] OR tsunami [Title/abstract])) |  |
| #2 | ("Acupuncture Therapy"[MeSH] OR "Acupuncture, Ear"[MeSH] OR "Acupuncture Points"[MeSH] OR "Acupuncture"[MeSH] OR "Electroacupuncture"[MeSH] OR "Meridians"[MeSH] OR acupuncture[Title/abstract] OR electroacupuncture[Title/abstract] OR electro-acupuncture[Title/abstract] OR acupoint*[Title/abstract]) |  |
| #3 | #1 AND #2 |  |

**EMBASE via Elsevier**

|  | Searches | Results |
| --- | --- | --- |
| #1 | (earthquake/exp OR earthquake OR tsunami/exp OR tsunami) |  |
| #2 | (‘acupuncture’/exp OR ‘acupuncture’ OR ‘acupuncture therapy’ OR ‘auricular acupuncture’/exp OR ‘auricular acupuncture’ OR ‘ear acupuncture’ OR ‘acupuncture point’/exp OR ‘acupuncture point’ OR ‘electroacupuncture’/exp OR ‘electroacupuncture’ OR ‘electro-acupuncture’ OR ‘body meridian’/exp OR ‘body meridian’ OR ‘acupoint’) |  |
| #3 | #1 AND #2 |  |

**CENTRAL**

|  | Searches | Results |
| --- | --- | --- |
| #1 | MeSH descriptor: [earthquake] explode all trees |  |
| #2 | (earthquake OR tsunami):ti,ab,kw |  |
| #3 | #1 OR #2 |  |
| #4 | MeSH descriptor: [Acupuncture] explode all trees |  |
| #5 | MeSH descriptor: [Acupuncture Therapy] explode all trees |  |
| #6 | MeSH descriptor: [Acupuncture, Ear] explode all trees |  |
| #7 | MeSH descriptor: [Electroacupuncture] explode all trees |  |
| #8 | (Acupuncture OR Pharmacopuncture OR Ear acupuncture OR Pharmacoacupuncture OR Electroacupuncture OR Acupotomy OR Acupotomies OR Ear acupuncture OR Auricular acupuncture):ti,ab,kw |  |
| #9 | #4 OR #5 OR #6 OR #7 OR #8 |  |
| #10 | #3 AND #9 in Trials |  |

**Web of Science**

|  | Searches | Results |
| --- | --- | --- |
| #1 | (TS=(earthquake) OR TS=(tsunami)) |  |
| #2 | (TS=(aupuncture therapy) OR TS=(acupuncture) OR TS=(acupuncture point) OR TS=(auricular acupuncture) OR TS=(ear acupuncture) OR TS=(electroacupuncture) OR TS=(electro-acupuncture) OR TS=(meridian) OR TS=(acupoint*)) |  |
| #3 | #1 AND #2 |  |

**Scopus**

|  | Searches | Results |
| --- | --- | --- |
| #1 | (TITLE-ABS-KEY (earthquake) OR TITLE-ABS-KEY (tsunami) |  |
| #2 | (TITLE-ABS-KEY (aupuncture therapy) OR TITLE-ABS-KEY (acupuncture) OR TITLE-ABS-KEY (acupuncture point) OR TITLE-ABS-KEY (auricular acupuncture) OR TITLE-ABS-KEY (ear acupuncture) OR TITLE-ABS-KEY (electroacupuncture) OR TITLE-ABS-KEY (electro-acupuncture) OR TITLE-ABS-KEY (meridian) OR TITLE-ABS-KEY (acupoint*)) |  |
| #3 | #1 AND #2 |  |

**AMED via EBSCO**

|  | Searches | Results |
| --- | --- | --- |
| #1 | (earthquake[TX] OR tsunami[TX]) |  |
| #2 | (“Acupuncture Therapy”[SU] OR “Acupuncture, Ear”[SU] OR “Acupuncture Points”[SU] OR Acupuncture[SU] OR Electroacupuncture[SU] OR Meridians[SU] OR acupuncture[TX] OR electroacupuncture[TX] OR electro-acupuncture[TX] OR acupoint*[TX]) |  |
| #3 | #1 AND #2 |  |

**CINAHL via EBSCO**

|  | Searches | Results |
| --- | --- | --- |
| #1 | (earthquake[TX] OR tsunami[TX]) |  |
| #2 | (“Acupuncture Therapy”[MH] OR “Acupuncture, Ear”[MH] OR “Acupuncture Points”[MH] OR Acupuncture[MH] OR Electroacupuncture[MH] OR Meridians[MH] OR acupuncture[TX] OR electroacupuncture[TX] OR electro-acupuncture[TX] OR acupoint*[TX]) |  |
| #3 | #1 AND #2 |  |

**PsycARTICLES via ProQuest**

|  | Searches | Results |
| --- | --- | --- |
| #1 | (earthquake OR tsunami) |  |
| #2 | mesh(Acupuncture Therapy) OR mesh(Acupuncture, Ear) OR mesh(Acupuncture Points) OR mesh(Acupuncture) OR mesh(Electroacupuncture) OR mesh(Meridians) OR ‘acupuncture’ OR ‘electroacupuncture’ OR ‘electro-acupuncture’ OR acupoint* |  |
| #3 | #1 AND #2 |  |

**OASIS**

|  | Searches | Results |
| --- | --- | --- |
| #1 | (지진 OR 해일) AND 침 |  |

**KCI**

|  | Searches | Results |
| --- | --- | --- |
| #1 | (지진 OR 해일) AND 침 |  |

**CNKI**

|  | Searches | Results |
| --- | --- | --- |
| #1 | (SU=''地震'+'海啸') AND (SU='acupuncture'+'针'+'鍼') |  |

**Wanfang data**

|  | Searches | Results |
| --- | --- | --- |
| #1 | 主题:(‘'地震'+'海啸') * 主题:("acupuncture" + "针" + "鍼") |  |

**VIP**

|  | Searches | Results |
| --- | --- | --- |
| #1 | (M=(地震 OR 海啸 (M=(acupuncture OR 针 OR 鍼)) |  |

**CiNii**

|  | Searches | Results |
| --- | --- | --- |
| #1 | (地震 OR 津波) AND (acupuncture OR 針 OR 鍼)) |  |
