# Supplementary material for: Effect of acupuncture on patients with major psychiatric disorder and related symptoms caused by earthquake exposure: Protocol for a scoping review of clinical studies
Source: PLoS One. 2023 Jan 27;18(1):e0281207. doi: 10.1371/journal.pone.0281207 (PMC9882882; doi:10.1371/journal.pone.0281207)
Supplement: S2 Table — (DOCX) [file pone.0281207.s002.docx]

S2 Table. Central characteristics of existing psychological therapies for PTSD

| Type of psychological therapy | | Description | Developer | The evidence of effectiveness | Benefits | Limitations |
| --- | --- | --- | --- | --- | --- | --- |
| TF-CBT | Prolonged exposure | Reducing anxiety and fear through confrontation of thoughts (imaginal exposure) or actual situations (in vivo exposure) related to the trauma that would otherwise provoke fear and avoidance | Drs. Tony Mannarino, Esther Deblinger, and Cohen developed and tested TF-CBT for traumatized children in 2003. TF-CBT was defined in the 2006 treatment manual “Treating Trauma and Traumatic Grief in Children and Adolescents” | Exposure therapy has a very strong evidence base | Helping patients reduce avoidance and maladaptive associations with the trauma. Helping patients learn how to cope with these until their anxiety levels lower and, ideally, become non-existent when confronted with the stimuli | High rates of patient nonresponse and dropout from treatment  High percentage of patients retaining PTSD diagnosis after treatment  Marginal benefits of leading PTSD treatments over other therapeutic techniques |
|  | Cognitive behavioral therapy | Modification of illogical and unrealistic interpretations of events that cause negative emotional reactions |  | There is robust evidence for the clinically important effect of the therapies broadly defined as TF-CBT | Helping people reconceptualize their understanding of traumatic experiences and their understanding of themselves and their ability to cope |  |
|  | Cognitive processing therapy | Combining writing a trauma narrative, which is read to the therapist (i.e., exposure), with cognitive therapy |  |  | Helping return a sense of control, self-confidence, and predictability to the patients and reduce escape and avoidance behaviors |  |
| EMDR | | Involving the patient moving his/her eyes back and forth (e.g., watching light bars or therapist’s fingers) during imaginal exposure and cognitive restructuring and stimulating the patient’s information processing to help corporate the targeted events as an adaptive memory. EMDR includes core elements of TF-CBT | EMDR was developed in the late 1980s when Francine Shapiro discovered a connection between eye movement and a decrease in the negative emotions associated with her own upsetting memories | EMDR appears equally effective as TF-CBT | Helping match the way you mentally process memories unconsciously to help reduce the amount of stress and anxiety related to the memories  EMDR can be freeing for clients who prefer not to share details about their traumatic experience because it does not require much talking, at least during the desensitization phase | Experts still cannot fully explain why it works, despite the evidence that it works  Limiting evidence of long-term efficacy |

PTSD, posttraumatic stress disorder; TF-CBT, trauma-focused cognitive behavioral therapy; EMDR, eye movement desensitization therapy.
